# Characteristics of immune clusters and cell abundance in patients with different subtypes of nonparoxysmal atrial fibrillation

**DOI:** 10.1038/s41598-022-26749-z

**Published:** 2023-01-18

**Authors:** Hangying Ying, Wenpu Guo, Pengcheng Yu, Hangyuan Qiu, Ruhong Jiang, Chenyang Jiang

**Affiliations:** Department of Cardiology, Key Laboratory of Cardiovascular Intervention and Regenerative Medicine of Zhejiang Province, Sir Run Run Shaw Hospital, Zhejiang University School of Medicine, Hangzhou, 310016 Zhejiang China

**Keywords:** Cardiovascular biology, Data mining, Microarrays

## Abstract

Atrial fibrillation (AF) is the most common sustained arrhythmia in clinical practice. Inflammation plays an important role in the initiation and perpetuation of AF. The present study was conducted to characterize immune clusters in nonparoxysmal AF and to distinguish immune subtypes of nonparoxysmal AF. Immune-related algorithms (CIBERSORT, ESTIMATE, and ssGSEA) were used to evaluate the immune cluster characterization and cell abundance, and multivariable logistics analysis was performed to determine the most relevant immune cells. We identified differentially expressed genes (DEGs) and used consensus clustering analysis to identify nonparoxysmal AF subtypes. Weighted gene coexpression network analysis (WGCNA) was used for finding highly correlated gene sets and attach to external sample traits. And it was conducted twice to identify the immune- and subtype- related modules. Finally, Metascape was used to compare the biological functions of the two nonparoxysmal AF subtypes we obtained. CytoHubba was used to identify the hub genes of these two subtypes. Based on the results of bioinformatics analysis, regulatory T cells, resting NK cells, active mast cells and neutrophils were considered to be closely related to nonparoxysmal AF. The brown module was identified as the most relevant module to the above immune cells by WGCNA. We identified two major nonparoxysmal AF subtypes by consensus clustering analysis and their enriched biological functions by Metascape. The hub genes are TYROBP, PTPRC, ITGB2, SPI1, PLEK, and CSF1R in permanent AF and JAM3, S100P, ARPC5, TRIM34, and GREB1L in persistent AF. This study revealed two major nonparoxysmal AF subtypes and eleven hub genes, which provide potential therapeutic targets for anti-inflammatory treatments of nonparoxysmal AF.

## Introduction

Atrial fibrillation (AF) is the most common cardiac arrhythmia in clinical practice. AF affects 1–2% of the general population worldwide, and the lifetime risk in Europe is 37% (34.3% to 39.6%)^1^. The main pathogenic mechanisms of AF include electrical remodeling, structural remodeling, calcium handling abnormalities and autonomic nervous system changes^2^. Recently, substantial evidence suggests that inflammation and its associated immune response are associated with the initiation and maintenance of AF, and the presence of AF could promote inflammation, leading to ‘AF begets AF’^3^. The infiltration of specific immune cells and the presence of inflammation markers could predict the onset and recurrence of AF in the general population, as well as in patients who undergo operative surgery^4^. Excessive inflammation and its inflammatory response could alter atrial electrophysiology and structural substrates^5,6^. Changes in calcium homeostasis caused by inflammation promote heterogeneous atrial conduction^3^, and these mechanisms lead to increased susceptibility to AF. Although inflammation takes part in the initiation and perpetuation of AF, the efficacy of anti-inflammatory treatment in the clinic is far from satisfactory. Like the varied efficacy of immunotherapy for different immunophenotypes in tumors, we speculated that there is varied efficacy of anti-inflammatory drugs based on the presence of different immune-related subtypes in AF.

**Figure 1:**
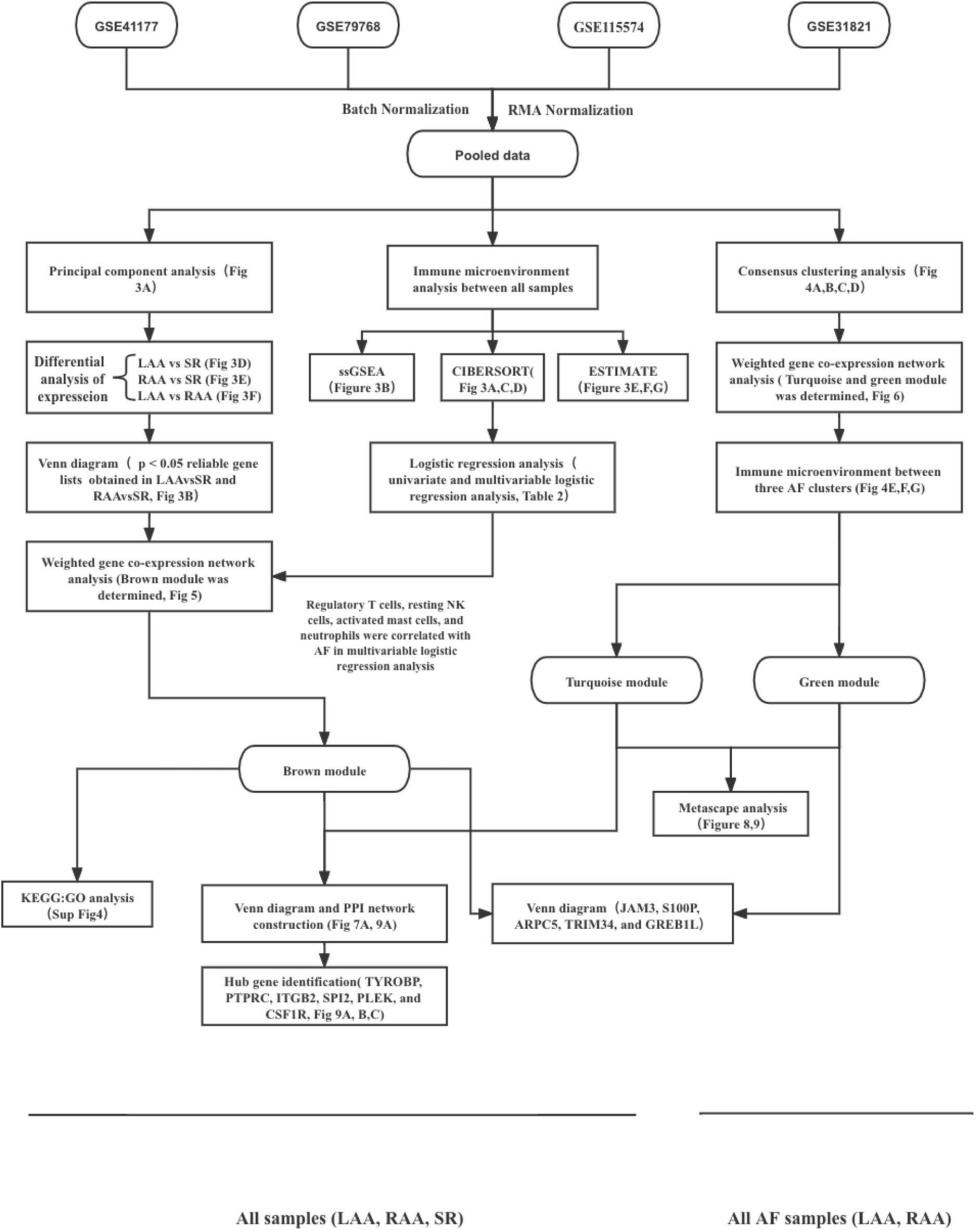
The analytical workflow.

Therefore, we performed this study using comprehensive bioinformatics analysis to further explore the relationship between inflammation and AF. In this study, immune-related algorithms were used to evaluate immune cluster characterization and cell abundance, and multivariable logistic regression analysis was performed to determine the most relevant immune cells. We identified differentially expressed genes (DEGs) and used consensus clustering analysis to identify nonparoxysmal AF subtypes. Weighted gene coexpression network analysis (WGCNA) was conducted twice to identify the immune- and subtype-related modules. Finally, Metascape was used to compare the biological functions of the two AF subtypes we obtained. CytoHubba was used to identify hub genes of these two AF subtypes. The whole workflow of this study was shown in Fig. 1.

## Methods

### Data availability and processing

The raw array data are available in the Gene Expression Omnibus database (GEO submission: GSE41177, GSE79768, GSE115574, and GSE31821, Table 1). In total, 41 left atrial appendage (LAA) samples and 21 right atrial appendage (RAA) samples from nonparoxysmal AF individuals were enrolled in this study. Forty-eight atrial appendages from sinus rhythm (SR) individuals served as controls. The raw data were preprocessed using the robust multi-array average (RMA) algorithm with the ‘affy’ package. The process includes background correcting, normalizing, and calculating expression (Supplemental Fig. 1B). The processed results were presented in log base 2 scale. The batch effect was removed using the ComBat function of the ‘sva’ package in R language (Supplemental Fig. 1C).

**Table 1:** Detail information for GEO databases.

Accession	Platform	Type of RNA	Organism	Number of samples	Time	Country	PMID
GSE115574	GPL570	Expression profiling by array	Homo sapiens	LAA and RAA from 15 persistent AF patients and 15 SR patients (30/30)	2018.6.11	Turkey	NA
GSE79768	GPL570	Expression profiling by array	Homo sapiens	LAA and RAA from 7 persistent AF patients and 6 SR patients (14/12)	2016.3.31	China Taiwan	27,494,721
GSE41177	GPL570	Expression profiling by array	Homo sapiens	16 LAA from persistent AF patients/3 left atrial appendages from SR patients (16/3)	2012.9.26	China Taiwan	23,183,193
GSE31821	GPL570	Expression profiling by array	Homo sapiens	4 AF auricle tissues/2 control auricle tissue (4/2)	2011.9.11	France	NA

### Immune cluster characterization and cell abundance

Bioinformatics algorithms (CIBERSORT, ESTIMATE, and ssGSEA) were used to evaluate the immune cluster characterization and cell abundance. CIBERSORT, a deconvolution algorithm, was used to assess the abundance of immune-related cells for each sample^7^. Pearson’s correlation was performed to assess the correlations between immune cell subsets. The correlation results are presented only for P < 0.05 in the correlation heatmap. The ESTIMATE algorithm was applied to compute the proportion of immune and stromal components in the immune microenvironment. Three parts of the scores were positively ﻿correlated with the proportion of stromal and immune cells and the sum of the first two^8^. Single sample gene-set enrichment analysis (ssGSEA) was used to classify gene sets with similar immune biological roles^9^. And ssGSEA was conducted by using ‘GSVA’ package^9^. A total of 681 immune-related genes were divided into 25 gene sets based on a well-known article^10^. The immune cluster characterization we applied consisted of innate immunity and adaptive immunity. The ssGSEA scores of each immune cell type were standardized, and pairwise comparisons were performed among the three groups.

### Logistics regression analysis

The presence of AF was designated a dependent variable, and the relative expression of immune cells was identified by CIBERSORT as an independent variable to conduct univariate logistic regression analysis. To prevent the influence of confounding factors, the variables with p ≤ 0.2 in the univariable logistics regression analysis were included in the follow-up multivariable logistics regression analysis with p < 0.05.

### Differential analysis of expressed genes

The ‘limma’ package in the R language was utilized to identify differentially expressed genes (DEGs) with an adjusted P < 0.05 and | logFC |> 0.5 among the three contrast matrices (LAA vs. SR, RAA vs. SR, and LAA vs. RAA)^11^. The results were visualized by heatmaps (‘pheatmap’ package) and volcano plots (‘limma’ package and ‘ggrepel’ package). To screen credible genes for further research, we merged the reliable gene lists (with only an adjusted P value < 0.05) of two contrast matrices (LAA vs. SR, RAA vs. SR).

### Consensus clustering analysis and principal component analysis (PCA)

Clustering analysis algorithms were performed with the goal of exploring hidden groupings in a large dataset. To dissect nonparoxysmal AF heterogeneity, unsupervised consensus clustering analysis, performed by ‘ConsensusClusterPlus’ package, was applied in all AF samples to define the subtype of nonparoxysmal AF patients and repeated 1000 times to evaluate the stability of results. The key operating parameters included 80% item resampling and a maximum evaluated k of 9. The PCA was used to assess the distinction between LAA, RAA, and SR and to validate the cluster results.

### Weighted correlation network analysis

The reliable gene lists (4790 genes) and the top 25% (5548 genes) of the variance in the merged database were selected for coexpression network analysis for immune cluster characterization and unsupervised clustering characterization. We used the ‘WGCNA’ package to select an appropriate soft-thresholding power β to achieve scale-free topology^12^. Then, the selected genes were clustered into modules, and each module was marked with different colors using the average linkage hierarchical clustering method. The minimum number of genes in each module was 100, and the threshold for module merging was 0.15 in immune cluster analysis and 0.25 in AF cluster analysis. In the first immune-related WGCNA, we used Pearson’s correlation method to calculate the correlation between each module and the relative expression of immune cells identified by CIBERSORT. The modules that are indicated by logistics results were selected for further analyses. In the second WGCNA of AF subtypes, we used Pearson’s correlation method to calculate the correlation between each module and clinical cluster generated by consensus cluster analysis.

### Functional enrichment analysis

The ‘clusterProfiler’ package was used to perform Gene Ontology (GO) and Kyoto Encyclopedia of Genes and Genomes (KEGG) pathway enrichment analyses^13,14^. Metascape (http://metascape.org/) was applied to investigate different biological functions between AF subtypes and to perform enrichment analysis among different gene sets^15^. An adjusted p < 0.05 was considered statistically significant.

### Construction of PPI network and hub gene identification

The intersection of selected modules (MEbrown and MEturquoise) was imported into the Search Tool for the Retrieval of Interacting Genes (STRING, v11.0) to generate the PPI network^16^. The results were visualized through Cytoscape software (v3.8.2). The CytoHubba plug-in was used to identify the hub genes through five local- or global-based algorithms^17^. The intersections of the five algorithms in CytoHubba were considered the hub genes in the PPI network.

### Statistical analysis

Most bioinformatic analyses were conducted in R software (4.0.5) with default statistical settings and cutoff values specified in the individual method sections. Data are expressed as the mean ± standard deviation. Statistical analyses were performed using GraphPad Prism software (version 9.0.0) and R software (4.0.5), and P < 0.05 was considered significantly different.

### Ethics statements

This article does not contain any studies with human participants performed by any of the authors. All methods were performed in accordance with the relevant guidelines and regulations.

## Results

### Immune cluster characterization and cell abundance

**Figure 2:**
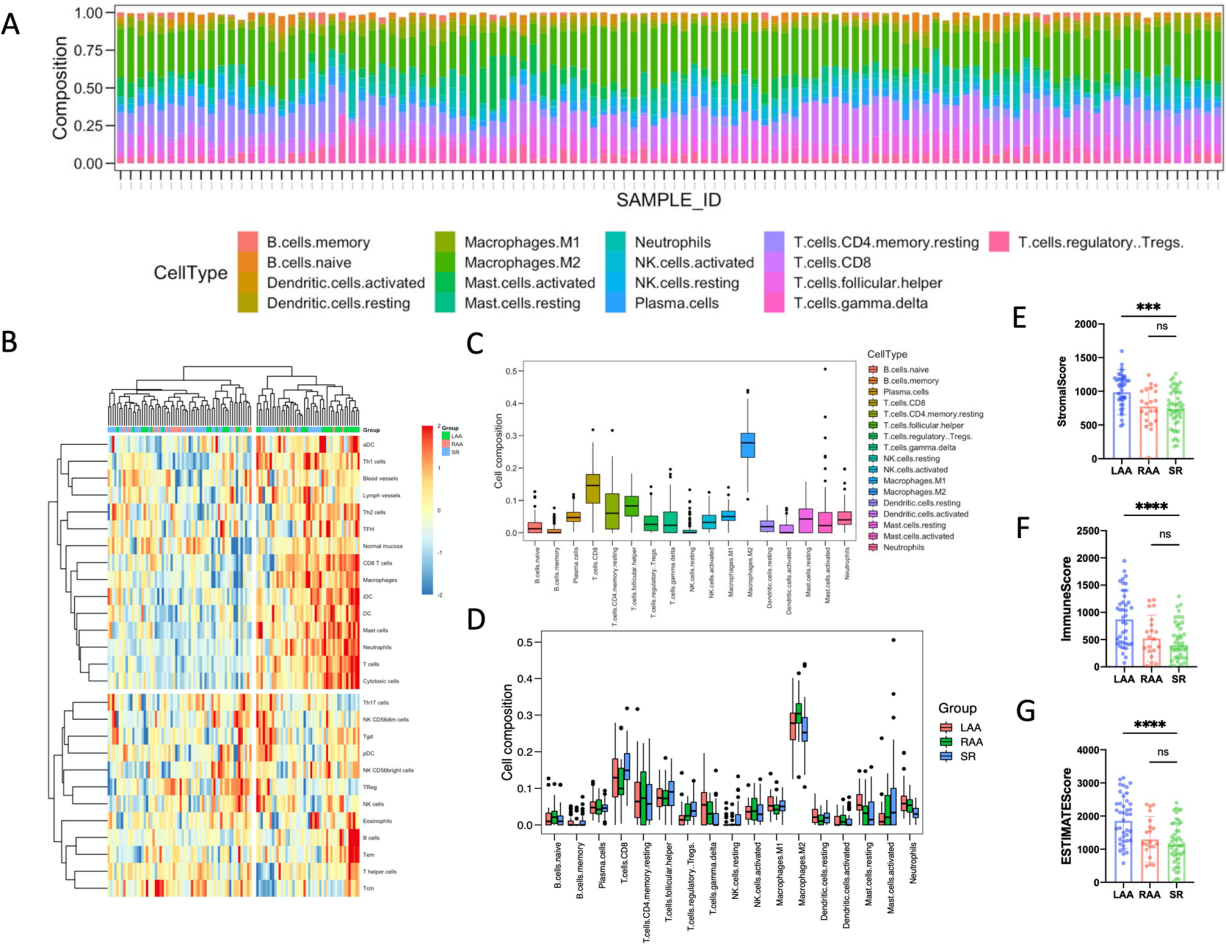
Immune cluster characterization and cell abundance. (**A**) Relative expression of 17 immune cell abundance stacked column charts in each sample estimated using CIBERSORT. (**B**) Immune cluster characterization heatmap using ssGSEA (‘GSVA’ package). (**C**) Immune cell abundance histogram of all included samples estimated using CIBERSORT. (**D**) Immune cell abundance histogram of different groups estimated using CIBERSORT. (**E**) Stromal score of three different groups using ESTIMATE. (**F**) Immune score of three different groups using ESTIMATE. (**G**) ESTIMATE score of three different groups using ESTIMATE. Data are presented as the mean ± SD. * p < 0.05, ** p < 0.01, *** p < 0.001, and **** p < 0.0001.

As shown in Fig. 2A,C, 17 types of immune cell subtypes were estimated in the included samples using CIBERSORT. M2 macrophages and T cells accounted for the majority of all types of immune abundances. B cells, NK cells and neutrophils represented a small minority. Further analyses showed that the abundance of regulatory T cells and activated mast cells gradually increased in the LAA, RAA and SR. Gamma delta T cells, resting mast cells, and neutrophils gradually decreased in the same location (Fig. 2D). Notably, resting NK cells were eliminated in AF samples (LAA and RAA), and it could be inferred that NK cells were not expressed in AF samples. As presented in Supplemental Fig. 2C, neutrophils were positively correlated with gamma delta T cells and negatively correlated with T regulatory cells. Activated mast cells were negatively correlated with resting mast cells. Analyses using ESTIMATE showed the distribution of immune/stromal scores at each site in AF and SR samples. The LAA samples from AF individuals had higher immune/stromal scores and reached statistical significance. There were no obvious differences between the RAA and SR groups (Fig. 2E–G). Interestingly, we found no differences in each immune cell type using ssGSEA (Supplemental Fig. 2A,B). AF samples (LAA and RAA) and SR samples were not clearly clustered into two categories but were evenly distributed in the two clusters. AF samples in these two categories have different immune characteristics (Fig. 2B). It is assumed that there are two different subtypes of nonparoxysmal AF.

### Logistics regression analysis

In univariate logistics regression analysis, CD8 T cells (p = 0.06), regulatory T cells (p = 0.01), gamma delta T cells (p = 0.01), resting NK cells (p = 0.01), M2 macrophages (p = 0.01), resting mast cells (p = 0.11), activated mast cells (p = 0.01) and neutrophils (p < 0.01) were included in the next multivariable logistics regression analysis. Regulatory T cells (p = 0.0495), resting NK cells (p = 0.039), and activated mast cells (p = 0.016) were negatively associated with AF, and neutrophils (p = 0.010) were significantly positively correlated with AF in multivariable logistics regression analysis (Table 2).

**Table 2:** Logistics regression analysis.

Univariate logistics regression		Multivariate logistics regression analysis		
Cell type	P value	OR	OR (95%CI)	P value
B cells naive	0.67			
B cells memory	0.94			
Plasma cells	0.24			
T cells CD8	0.06	3.07 × 10^−4^	(3.89 × 10^−8^, 1.20)	0.06343
T cells CD4 memory resting	0.65			
T cells follicular helper	0.66			
T cells regulatory Tregs	0.01	2.09 × 10^−8^	(1.73 × 10^−16^, 8.02 × 10^−1^)	0.04954*
T cells gamma delta	0.01	2.59 × 10^−1^	(1.51 × 10^−7^, 7.01 × 10^5^)	0.85340
NK cells resting	0.01	7.52 × 10^−13^	(6.31 × 10^−26^, 1.22 × 10^−2^)	0.03856*
NK cells activated	0.80			
Macrophages M1	0.67			
Macrophages M2	0.06	1.79 × 10^1^	(3.42 × 10^−3^, 1.23 × 10^5^)	0.51019
Dendritic cells resting	0.84			
Dendritic cells activated	0.90			
Mast cells resting	0.11	1.52 × 10^−3^	(3.51 × 10^−10^, 2.88 × 10^3^)	0.38579
Mast cells activated	0.01	2.49 × 10^−7^	(2.05 × 10^−13^, 1.43 × 10^−2^)	0.01566*
Neutrophils	0.00	1.08 × 10^12^	(5.53 × 10^3^, 1.24 × 10^22^)	0.00967*

### Principal component analysis and differential analysis of expressed genes

**Figure 3:**
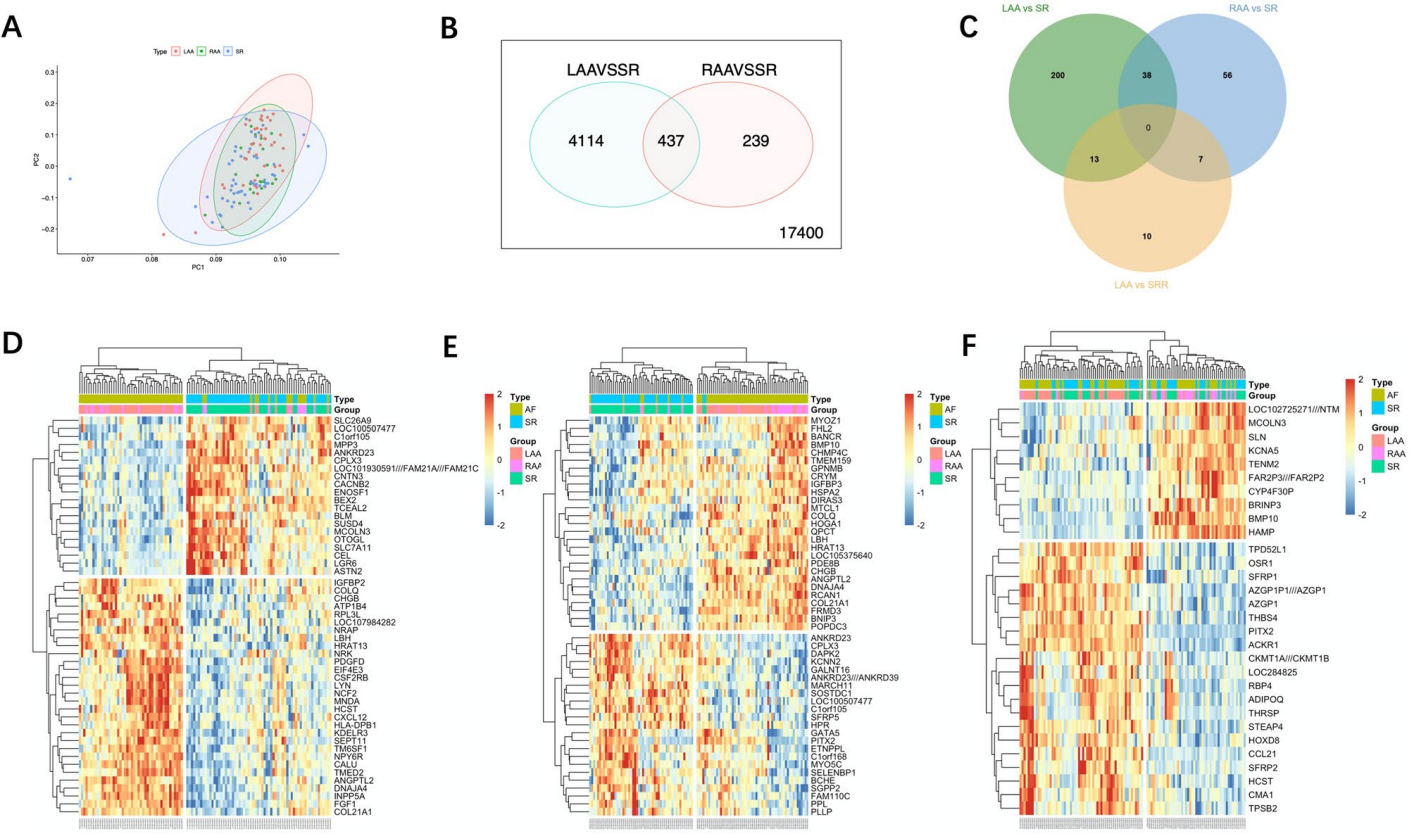
Principal component analysis and differential analysis of expressed genes. (**A**) Principal component analysis. (**B**) Venn plot of two contrast matrices (LAA vs. SR, RAA vs. SR). The numbers shown in circles are the adjusted P value < 0.05. (**C**) Venn plot of DEGs of three contrast matrices (LAA vs. SR, RAA vs. SR, and LAA vs. RAA). (**D**–**F**) Heatmap of DEGs (‘pheartmap’ package). (**D**) LAA vs. SR. (**E**) RAA vs. SR. (**F**) LAA vs. RAA.

As presented in Fig. 3A, there was no appreciable difference between RAA and SR. In differential expression analysis, we identified 251 DEGs in the ‘LAA vs. SR’ matrix, 101 DEGs in the ‘RAA vs. SR’ matrix and 30 DEGs in the ‘LAA vs. RAA’ matrix. There were no common DEGs among these three contrast matrices (Fig. 3C). DEGs are presented as a heatmap (Fig. 3D–F) and volcano diagram (Supplemental Fig. 3A–C). Similarly, AF samples (LAA and RAA) and SR samples were not clearly clustered into two categories and were evenly distributed in the two clusters (Fig. 3F). It could be speculated that differential analysis of the expression of genes is not an appropriate method for distinguishing nonparoxysmal AF subtypes. Significant differences in PITX2, BMP10, and HAMP are shown in Supplemental Fig. 3C. We obtained a reliable gene list by merging two contrast matrices for further research (Fig. 3B).

### Identification of nonparoxysmal AF subtypes and their immune environment

**Figure 4:**
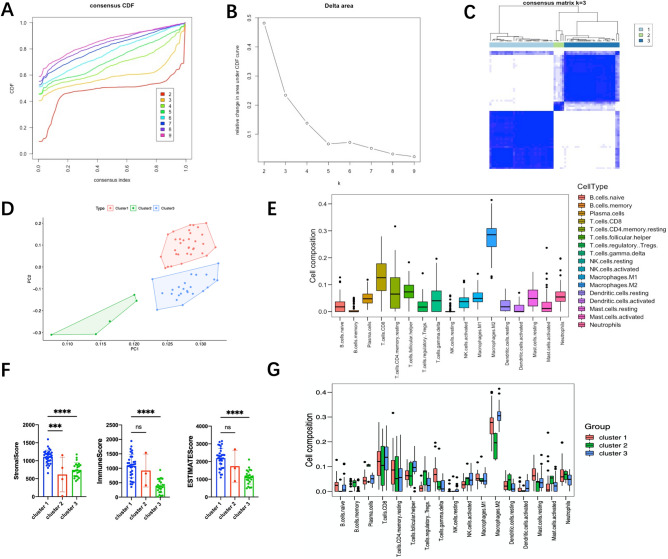
Identification of nonparoxysmal AF subtypes and their immune environment. (**A**) Cumulative distribution function (CDF) plots. (**B**) Delta area plot. (**C**) Consensus matrix plot (‘ConsensusClusterPlus’ package). (**D**) Principal component analysis of AF samples. (**E**) Immune cell abundance histogram in AF samples estimated using CIBERSORT. (**F**) Stromal/immune scores in three different clusters using ESTIMATE. (**G**) Immune cell abundance histogram of different clusters of AF samples estimated using CIBERSORT.

Consensus clustering analysis was performed to identify the subtypes of nonparoxysmal AF. According to the cumulative distribution function (CDF) plots (Fig. 4A) and delta area plot (Fig. 4B), we clustered AF samples into 3 groups. The boundaries of these 3 clusters were clear. Clusters 1 and 3 accounted for the vast majority of nonparoxysmal AF samples (Fig. 4C,D), and we turned our attention to these two clusters. As demonstrated previously in Fig. 2C, M2 macrophages and T cells accounted for the majority of all types of immune abundance. B cells, NK cells and neutrophils represented a small minority (Fig. 4E). Further analyses of immune cluster characterization revealed no significant differences in neutrophils and resting mast cells among clusters 1 and 3. Gamma delta T cells were higher in cluster 1 than in cluster 3 (Fig. 4G). For the ESTIMATE scores, the cluster 1 samples had higher immune/stromal scores than the cluster 3 samples and reached statistical significance (Fig. 4F).

### Construction of the weighted coexpression network and identification of key modules

**Figure 5:**
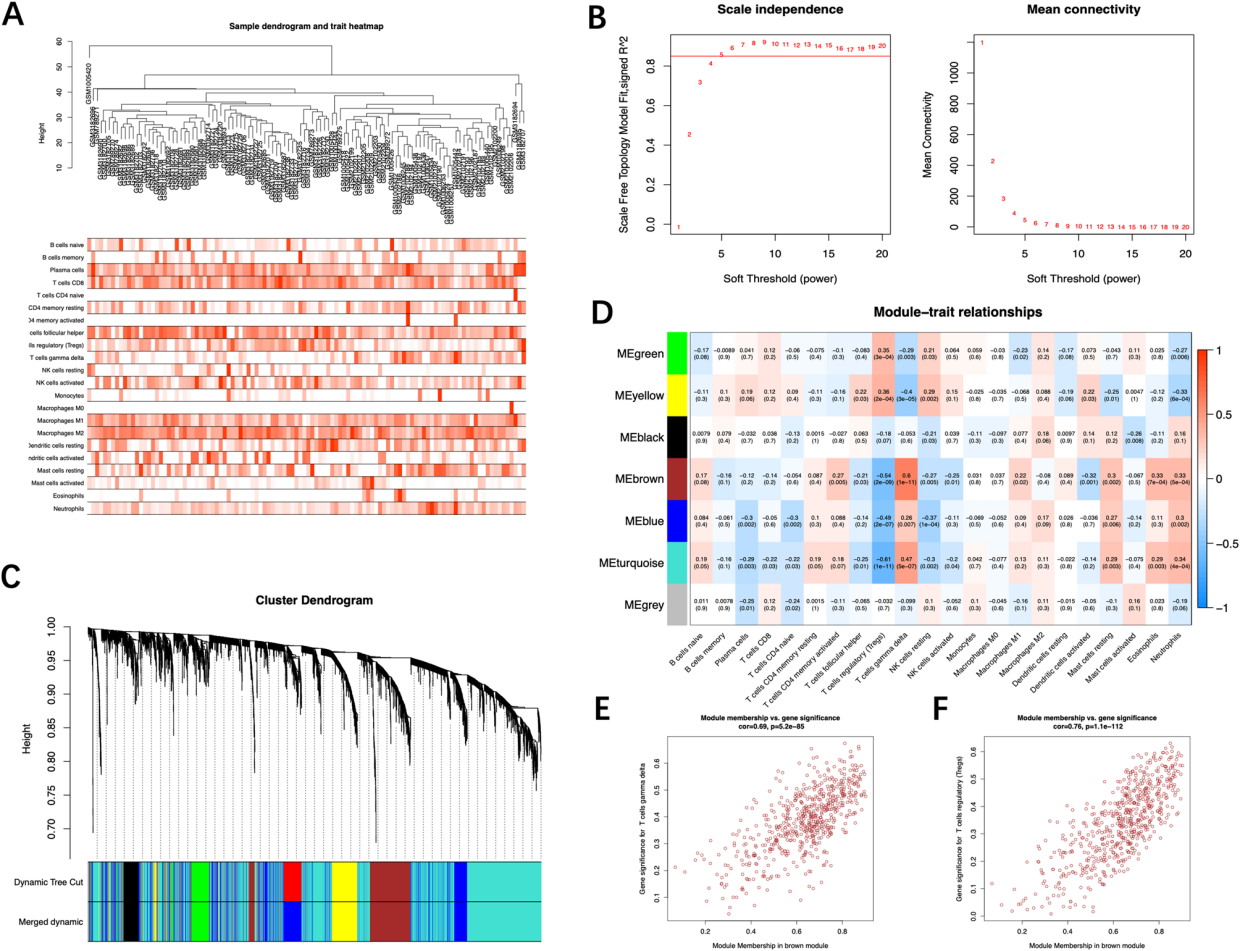
Construction of the weighted coexpression network and identification of key modules (‘WGCNA’ package). (**A**) Sample dendrogram and trait heatmap. (**B**) The selection of the soft-thresholding power β. The red line was set at 0.85, and the soft-thresholding power β was 5. (**C**) Dendrogram of all the differentially expressed genes. (**D**) Module-trait relationships in the constructed network. According to the logistics results, the brown and turquoise modules are thought to be closely related to the presence of atrial fibrillation. (**E**,**F**) Scatter diagrams for module membership vs. gene significance of the disease state in the brown module.

**Figure 6:**
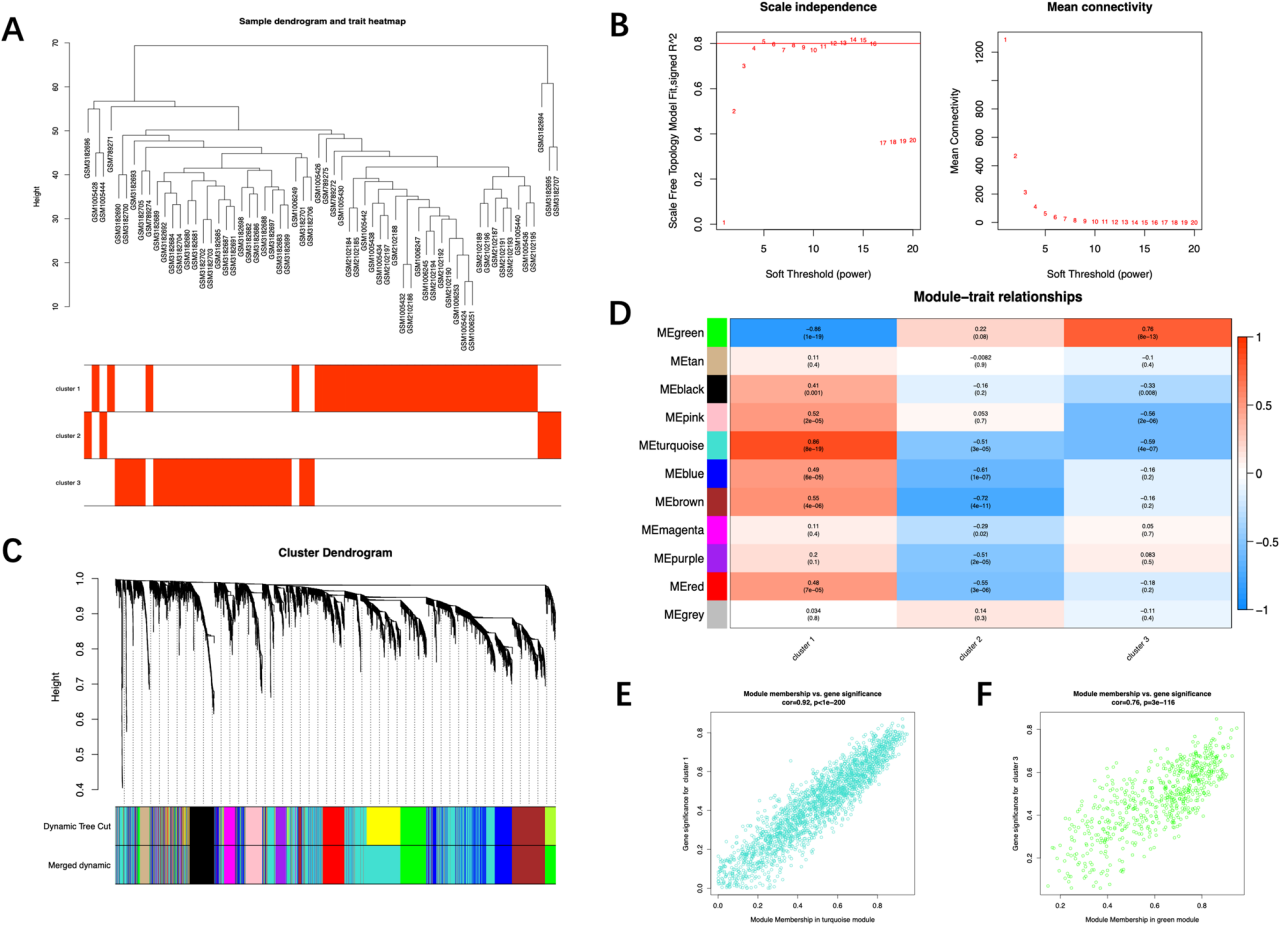
Construction of the weighted coexpression network and identification of key modules (‘WGCNA’ package). (**A**) Sample dendrogram and trait heatmap. The vast majority of samples were split into clusters 1 and 3. (**B**) The selection of the soft-thresholding power β. The red line was set at 0.80, and the soft-thresholding power β was 5. (**C**) Dendrogram of all the differentially expressed genes. (**D**) Module-trait relationships in the constructed network. The correlation in the green and turquoise modules was completely opposite between cluster 1 and cluster 3. (**E**) Scatter diagrams for module membership vs. gene significance of the disease state in the turquoise module. (**F**) Scatter diagrams for module membership vs. gene significance of the disease state in the green module.

In this study, WGCNA was conducted twice for different purposes. In the first analysis, based on the reliable gene lists and cutoff value (cutHeight = 40, Fig. 5A), a total of 104 samples and 4790 genes were included in the WGCNA. Soft-threshold β = 5 was applied to construct a gene coexpression network (Fig. 5B). According to logistics results and the correlation between module eigengenes (MEs) and immune cluster characterization, the brown and turquoise modules are thought to be closely related to the presence of AF (Fig. 5D). Because of the larger number of genes in the turquoise module (2748 genes, Fig. 5C), brown module genes were included in subsequent analyses. The brown modules showed a high positive correlation with T cells gamma delta (cor = 0.62; P = 5.2 × 10^−85^, Fig. 5E) and T cells regulatory (cor = 0.76; P = 1.1 × 10^−112^, Fig. 5F). In the second WGCNA, the top 25% (5548 genes) of the variance in the 62 AF samples was included in the WGCNA. Soft-threshold β = 5 was applied to construct a gene coexpression network (Fig. 6B) and most of genes are in turquoise module (Fig. 6C). As shown in Fig. 6A, most samples from AF individuals were split into two different clusters, namely, clusters 1 and 3. It is striking that all samples included in cluster 3 come from individuals with permanent AF, and samples included in cluster 1 belong to all persistent AF and very few permanent AF patients. Most modules in cluster 1 showed a high positive correlation with the generated cluster, and most modules in cluster 3 were negatively associated with the generated cluster. Notably, the correlation in the green and turquoise modules was completely opposite between cluster 1 and cluster 3 (Fig. 6D). The turquoise module showed a high positive correlation with cluster 1(cor = 0.92; P < 1.0 × 10^−200^, Fig. 6E) and green module was associated with cluster 3 (cor = 0.76; P = 3.0 × 10^−116^, Fig. 6F). These data suggest that gene sets from these two modules might perform distinct biological functions.

### Functional enrichment analysis

Functional enrichment analysis of the brown module in the first WGCNA offered an immune-related biological understanding of AF. The GO terms (Supplemental Fig. 4A) for biological processes (BP) were enriched mainly in leukocyte cell–cell adhesion, leukocyte proliferation and mononuclear cell proliferation. The molecular functions (MF) included immune receptor activity, antigen binding, and cytokine receptor activity (Supplemental Fig. 4B). The cellular components (CC) included the external side of the plasma membrane, secretory granule membrane and membrane raft (Supplemental Fig. 4C). For KEGG pathway enrichment analysis (Supplemental Fig. 4D), the brown module was significantly related to cytokine-cytokine receptor interactions, chemokine signaling pathways and Epstein-Barr virus infection.

### Metascape analysis

**Figure 7:**
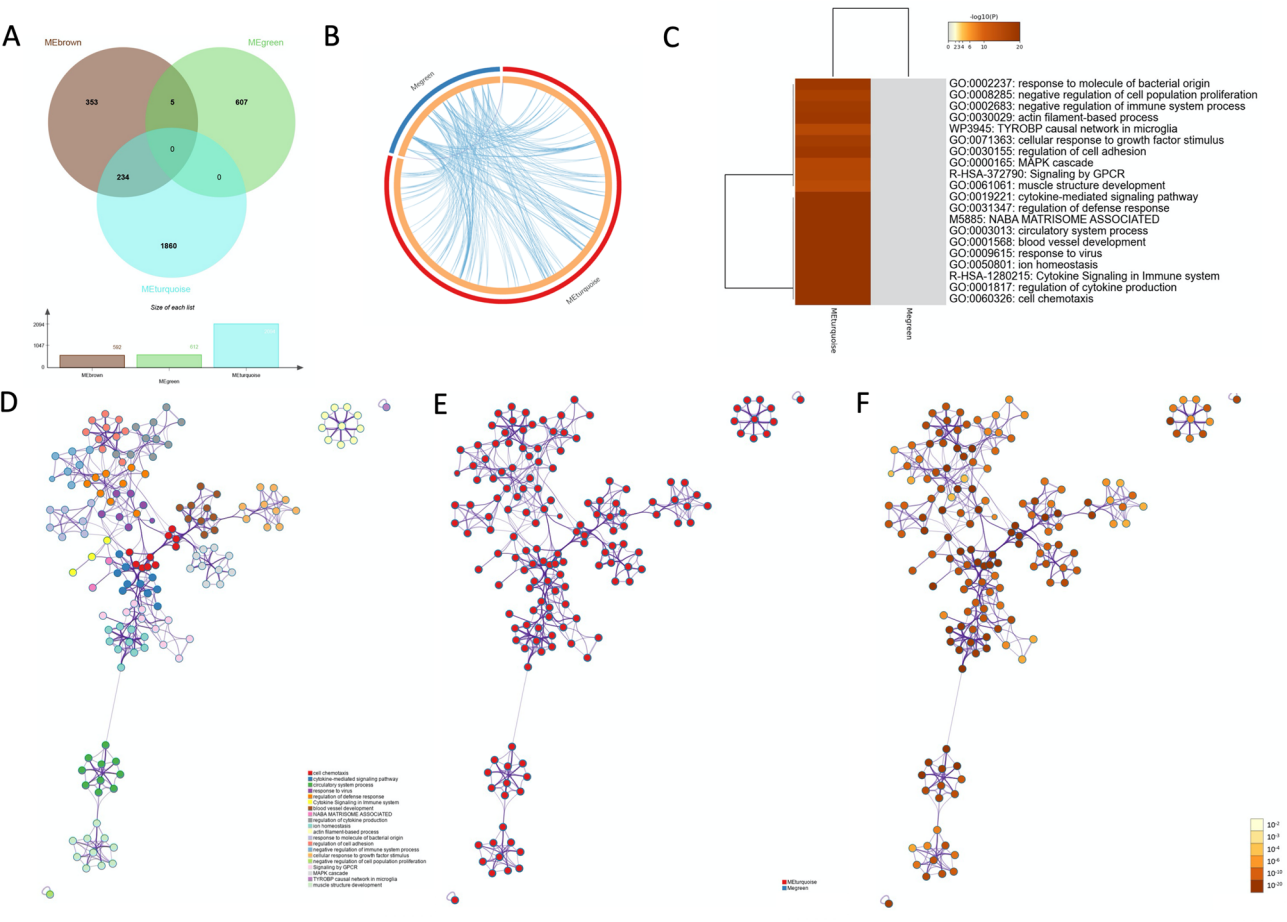
Pathway enrichment analysis of AF subtype (Metascape (http://metascape.org/)). (**A**) Venn diagram of the three modules. Five common genes (JAM3, S100P, ARPC5, TRIM34, and GREB1L) were considered hub genes in one AF subtype. (**B**) Circos plot of the green and turquoise modules. Blue lines link the different genes and where they fall within the same ontology term. (**C**) Pathway enrichment analysis of the green and turquoise modules. Biological functions that were enriched in the top 20 genes with the smallest p value. (**D**–**F**) The enrichment network of the green and turquoise modules. (**D**) The nodes of the network are displayed as pies, and each pie sector is proportional to the number of hits originating from a gene list. (**E**) Different colors represent various enrichment pathways of the gene list in the turquoise module. (**F**) Different shades of color represent the degree of enrichment, the darker the color represents the higher the degree of enrichment.

To disclose the biological functional properties associated with the AF subtypes (cluster 1, cluster 3), Metascape was conducted with the green and turquoise modules. As presented in Fig. 7B, blue links indicate the amount of functional overlap among these two gene lists. There are several common biological functions between the green and turquoise modules. In the turquoise module, which is strongly positively correlated with cluster 1 and negatively correlated with cluster 3, the GO terms GO:0060326: cell chemotaxis, GO:0019221: cytokine-mediated signaling pathway, GO:0050801: ion homeostasis and the WikiPathways term WP: TYROBP causal network were enriched among the top 20 terms with the smallest p value. Similar pathways were not enriched in the green module (Fig. 7C). For better concise presentation of the correlation between the green and turquoise modules, the subset of representative terms was selected and visualized within Cytospace. Consistent with Fig. 7C, the top 20 biological functions with the smallest p value enrichment network were found in the turquoise module results (Fig. 7E,F). The major enrichment pathways were cell chemotaxis, cytokine-mediated signaling pathways, ion homeostasis and the TYROBP causal network (Fig. 7D).

**Figure 8:**
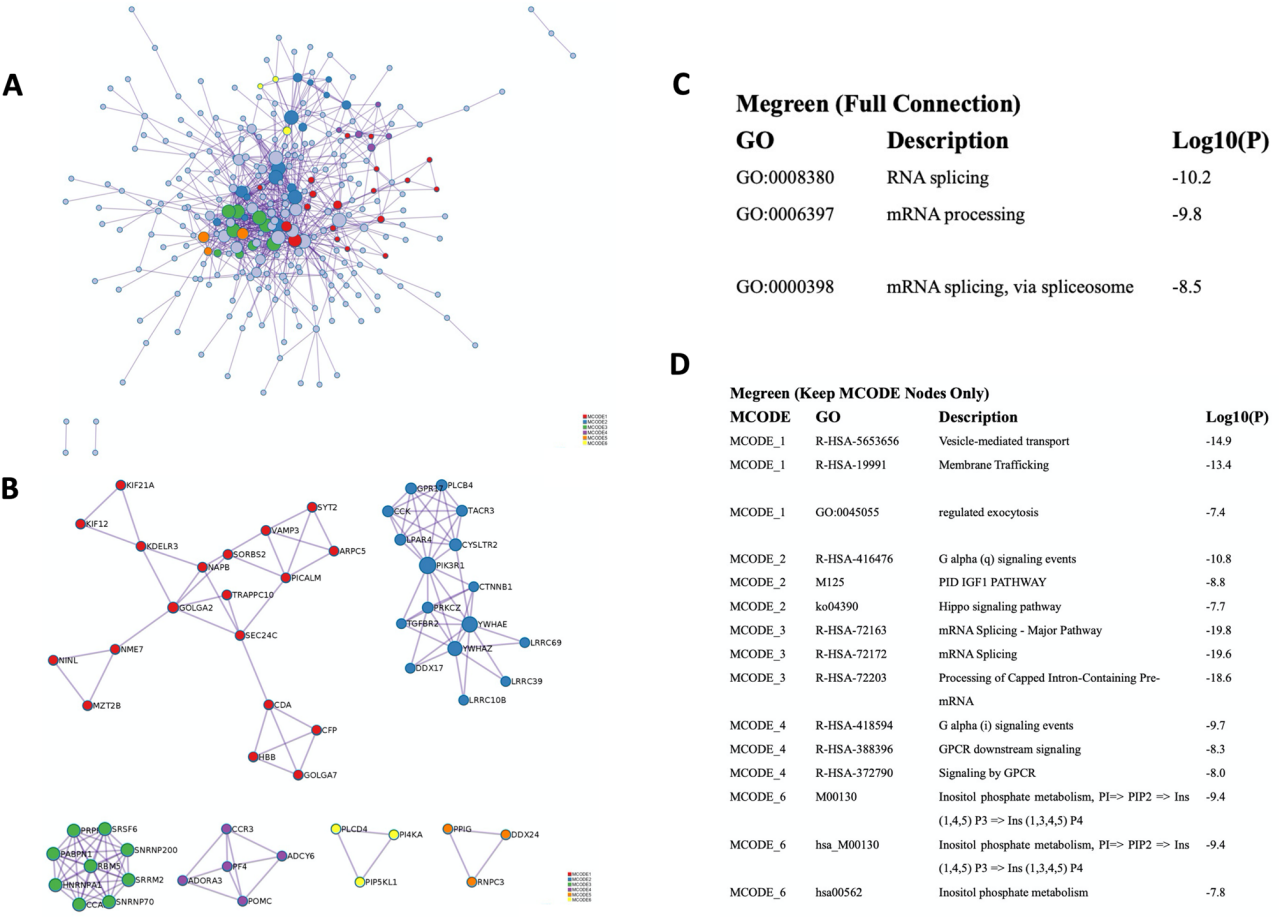
Pathway enrichment analysis of the green module (Metascape (http://metascape.org/)). (**A**) Protein–protein interaction (PPI) network of the green module. (**B**) GO enrichment analysis for PPI network and its MCODE network components. (**C**) Top three best p-value terms retained from GO enrichment analysis for original PPI network and its MCODE network. (**D**) Top biological terms etained from GO enrichment analysis for only MCODE nodes network.

Based on some understanding of the biological function of the turquoise module, we next focused on the green module. The PPI network of the green module was constructed, and the significant clusters were identified via the MCODE plugin. A total of 275 nodes and 730 edges were generated with the PPI network (Fig. 8A), and 6 clusters were screened out (Fig. 8B). In addition, we performed PPI enrichment analysis from full nodes and only MCODE nodes. In full node enrichment analysis, the GO terms GO:0008380: RNA splicing, GO:0006397: mRNA processing, and GO:0000398: mRNA splicing via spliceosome were enriched (Fig. 8C), and the results of MCODE node analysis are shown in Fig. 8D.

### Construction of the PPI network and hub gene identification

**Figure 9:**
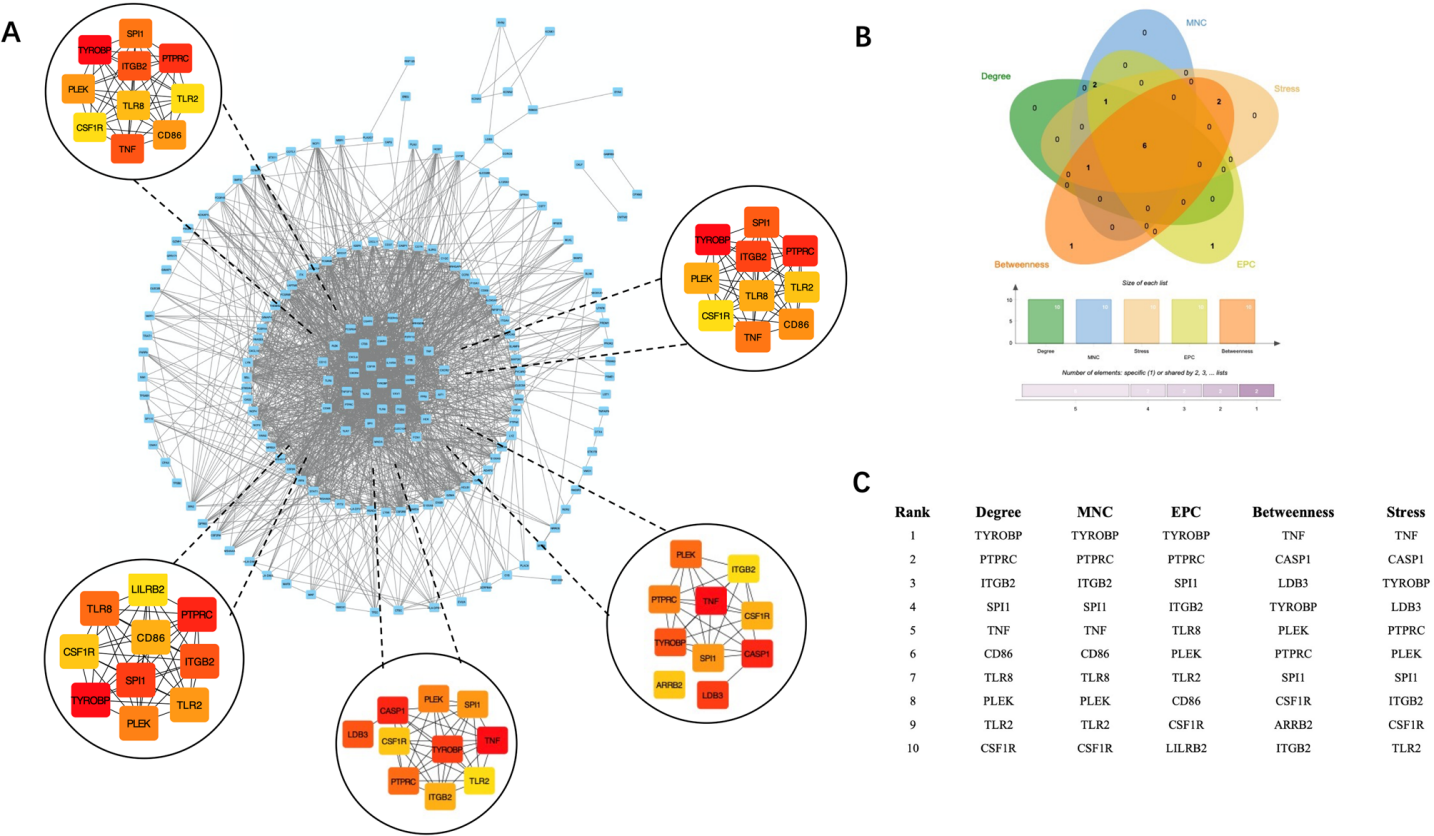
Identification of hub genes. (**A**) Visualization of the protein–protein interaction (PPI) network of the coincident part of two modules (MEbrown and MEturquoise) and visualization results of five algorithms of CytoHubba (STRING (v11.0) and Cytoscape software (v3.8.2)). (**B**) Venn diagram among five algorithms of CytoHubba. The intersection presented the hub genes (TYROBP, PTPRC, ITGB2, SPI1, PLEK, and CSF1R) determined by five algorithms. (**C**) Top ten hub genes identified by five algorithms, including Degree, MNC, EPC, Betweenness, and Stress.

To identify the hub genes in two different AF subtypes. A Venn diagram was applied to locate the intersection of the three modules selected from the WGCNA. No gene was found in the intersection, and only five genes were found in common in the green and brown modules (Fig. 7A). Therefore, five common genes (JAM3, S100P, ARPC5, TRIM34, and GREB1L) were considered hub genes in one AF subtype. The intersection of selected modules (MEbrown and MEturquoise) was used to construct the PPI network. Altogether, 176 nodes and 1741 edges were generated with the PPI network (Fig. 9A). Five algorithms of CytoHubba, including Degree, MNC, EPC, Betweenness, and Stress, were used to identify the top ten hub genes in each algorithm (Fig. 9C). TYROBP, PTPRC, ITGB2, SPI1, PLEK, and CSF1R were identified as hub genes in the other subtype (Fig. 9B).

## Discussion

Inflammation and its immune response play a significant role in the initiation and perpetuation of AF. Inflammation pathways are associated with cardiac structural and electrical remodeling and thrombogenesis^3^. However, the situation differs among atria. In a prospective-analysis study, the left atrial function index was associated with the incidence of AF. AF is considered a left atrial disease with subsequent changes occurring in the right atrium^18^. Similarly, recent transcriptomic and proteomic analyses revealed that the expression program in the left atrium had undergone a significant change compared with that in the right atrium^19^. Although inflammation has been closely correlated with AF, the efficacy of anti-inflammatory treatments in the clinic is far from satisfactory. Cortisol is the most widely prescribed drug and is a dose-dependent anti-inflammatory medicine^20^. Low-dose hydrocortisone could prevent AF recurrence after radiofrequency catheter ablation^21^. However, high-dose cortisol did not bring more benefits but rather side effects^20^. Statins, which serve as anti-inflammatory drugs ﻿inhibiting NOX2-NADPH oxidases, are similarly effective in preventing AF recurrence but are not currently used in treatment and management^22^. Like the varied efficacy of immunotherapy for different immune subtypes in tumors, we speculated that the varied efficacy of anti-inflammatory drugs was due to the presence of different immune-related subtypes in nonparoxysmal AF. Therefore, we performed this study using ﻿comprehensive bioinformatics analysis to further explore the relationship between inflammation and AF and identify the subtypes of nonparoxysmal AF. Interestingly, two nonparoxysmal AF subtypes we obtained are permanent and persistent AF. Samples included in cluster 1 belong to all persistent AF and very few permanent AF patients, and all samples included in cluster 3 come from individuals with permanent AF. In clinical practice, anti-arrhythmic drugs and ablation are applied in persistent AF, and rate control is only conducted in permanent AF^23^. It is well known that there might be a longer duration of AF, and a worse cardiac state who suffer permanent AF^23^. We speculated that the heart tissue in permanent AF exhibited a greater degree of inflammation than persistent patients. However, further analyses revealed that gamma delta T cells were higher in cluster 1 than in cluster 3, and the cluster 1 samples had higher immune/stromal scores than the cluster 3 samples in ESTIMATE. It indicated that there was more active inflammation during the course of persistent AF. According to results of Metascape, cluster 1 (persistent AF) was strongly positively correlated the GO terms GO:0060326: cell chemotaxis, GO: 0019221: cytokine-mediated signaling pathway, GO:0050801: ion homeostasis. And the cluster 3 (persistent AF) was associated with GO:0008380: RNA splicing, GO:0006397: mRNA processing, and GO:0000398: mRNA splicing. In conclusion, our results demonstrated that active inflammation promoted electrical and structural remodeling in progression of AF. However, when persistent AF turned to permanent AF, the inflammation remained a low and steady level.

Similarly, our results shed new light on the extent of inflammation and AF. In the immune characterization evaluation among different atria, we first identified the most relevant immune cell types by using CIBERSORT and logistics analysis (Table 2). We found that regulatory T cells (p = 0.0495), resting NK cells (p = 0.039), activated mast cells (p = 0.016), and neutrophils (p = 0.010) were significantly correlated with AF. These results supported the previous evidence. Activated mast cells exert structural remodeling actions by releasing profibrotic mediators^24^, and activated mast cells take part in electromechanical remodeling of myocytes mediated by platelet-derived growth factor^25^. Neutrophils, especially polymorphonuclear neutrophils, are believed to be causal factors for AF development and maintenance^4^. Peroxidases such as myeloperoxidase (MPO) and reactive oxygen species (ROS) mainly come from neutrophils and have been shown to propagate atrial remodeling^26^. Regulatory T cells were reported to attenuate ventricular remodeling induced by Angiotensin II in mice^27^. However, the role of natural killer cells in AF has not been reported thus far. Natural killer cells are proposed to be proinflammatory immune cells and are associated with immune activation and cytokine production^28^. Using the quantitative algorithm ESTIMATE, we found that LAA samples from AF individuals had higher immune/stromal scores and reached statistical significance. There were no obvious differences between the RAA and SR groups (Fig. 2E–G). Similarly, in the PCA among atria, we found that there was no significant difference between RAA and SR (Fig. 3A). This finding provides supporting evidence that AF is a left atrial disease with subsequent changes occurring in the right atrium. Overall, no significant difference was detected between AF (LAA and RAA) and SR samples (Fig. 3A). This result supports the views that distinct molecular changes occur rapidly in progression to the early stage of persistent AF, and subtle molecular changes occur 1 year after persistent AF^19^. As shown in Fig. 2 and Supplemental Fig. 2, we found that most major immune cells were present in both the LAA, RAA, and SR. Interestingly, we found no differences in each immune cell type using ssGSEA (Supplemental Fig. 2A,B). AF samples (LAA and RAA) and SR samples were not clearly clustered into two categories but were evenly distributed in the two clusters. AF samples in these two categories have different immune characteristics (Fig. 2B). We speculated that there might be two major subtypes in nonparoxysmal AF. Then, we obtained the most relevant gene module by using the WGCNA (Fig. 4D, brown module).

Likewise, a uniform distribution was similarly observed in the differential analysis of expressed genes (Fig. 3F). This finding further supported our hypothesis of the presence of subtypes in nonparoxysmal AF. Meanwhile, we identified PITX2, BMP10, and HAMP as significant DEGs with |logFC|> 2.0 in the ‘LAA vs. RAA’ matrix. PITX2 is one of the most important genes associated with AF by genome-wide association studies (GWAS), and its expression is largely limited in the left atrium^29^. BMP10 is mainly regulated by PITX2 and is confined to the right atrium. The plasma concentrations of PITX2 and BMP10 could predict the risk of recurrent AF after ablation^30^. The hepcidin gene (HAMP) is involved in the regulation of iron homeostasis, and mutation of HAMP causes cardiomyopathy and heart failure^31^. Although there is no direct clinical evidence to prove the relationship between HAMP and AF, it has been stated that the risk of AF increased stepwise with increasing ferritin concentration^32^.

Here, we conducted consensus clustering analysis and first obtained two major subtypes of nonparoxysmal AF. Cluster 1 and cluster 3 showed opposite gene profiles in the WGCNA modules. In particular, the correlation in the green and turquoise modules was completely opposite between cluster 1 and cluster 3 (Fig. 7D). These data suggest that gene sets from these two modules might perform distinct biological functions. Therefore, Metascape was used to disclose the biological functional properties associated with the AF subtypes (cluster 1 and cluster 3). We did not find enrichment of the same biological functions based on the top 20 genes with the smallest p values (Fig. 7C). In the turquoise module, cell chemotaxis, cytokine-mediated signaling pathways, ion homeostasis and the TYROBP causal network were enriched (Fig. 7D). In the green module, the pathways were associated with RNA splicing and mRNA processing (Fig. 8). In addition to the identification of subtypes, we also identified hub genes associated with both nonparoxysmal AF subtypes and immune cluster characterization. We obtained five common genes (JAM3, S100P, ARPC5, TRIM34, and GREB1L) as hub genes in the permanent AF subtype, and TYROBP, PTPRC, ITGB2, SPI1, PLEK, and CSF1R were determined to be hub genes in the persistent AF subtype.

In permanent AF subtype, five hub genes are closely related to cardiac structure, cytokine signaling in immune system, and ion homeostasis. JAM3 (junctional adhesion molecule 3) is a protein coding gene, playing a crucial role in the hypoplastic left heart^33^ and Tetralogy of Fallot^34^. JAM3 is associated with extracellular matrix organization and responses to elevated platelet cytosolic Ca^2+^^33,34^. S100P (s100 calcium binding protein p) is a member of the S100 family of proteins which relates to calcium ion binding and calcium-dependent protein binding^35^. S100P serves as a calcium sensor and contribute to cellular calcium signaling^35^. As for ARPC5 (actin related protein 2/3 complex subunit 5), it takes part in actin filament binding and serves as a structural constituent of cytoskeleton. When face to DNA damage, ARPC5 mediates the formation of branched actin networks in the cytoplasm and promote the movement of double-strand breaks^36^. TRIM34 (tripartite motif containing 34) is a member of the tripartite motif (TRIM) family. The expression of TRIM34 is up-regulated by interferon and it is believed in joining the defense against infections^37^. GREB1L (GREB1 like protein) is a protein-coding gene, which is involved in early metanephros and genital. Recent study revealed that GREB1L is correlated with immune cell infiltration in lung cancer^38^.

In persistent AF subtype, six hub genes are strongly correlated to immune signaling pathway. TYROBP is mainly expressed on immune cells, and it affects the immune response by modulating the immune cell functions^39^. Similarly, PTPRC (CD45) is a common antigen of leucocytes and is expressed on almost all haematopoietic cells except for mature erythrocytes. Disruption of CD45 would lead to immunodeficiency, autoimmunity, or malignancy^40^. ITGB2 (CD18) encodes an integrin beta chain and the encoded protein plays an important role in immune response. Loss of ITGB2 results in leukocyte adhesion deficiency^39,41^. Previous study found activated ITGB2 transcription regulated macrophage trafficking and contributed to the pathogenesis of cardiac hypertrophy in mice heart^41^. SPI1(Spi-1 proto-oncogene) encodes a transcription factor that activates gene expression during myeloid and B-lymphoid cell development. Interestingly, active SPI1 binds to RNA and may modulate pre-mRNA splicing^42^. CSF1R (CD115) is the receptor for colony stimulating factor 1. The CSF1 is a cytokine which controls the production, differentiation, and function of macrophages. Deletion of a CSF1R enhancer disrupts development of tissue macrophage populations^43^. As for PLEK (Pleckstrin), it is a protein coding gene involved in response to elevated platelet cytosolic Ca^2+^ and TYROBP causal network in microglia. PLEK is served as a substrate for protein kinase C (PKC) enzymes, and it takes part in cytoskeletal reorganization, promoting cell–cell adhesion, and migration^44^.

Our concerted use of these bioinformatics analyses identified two subtypes of nonparoxysmal AF and hub genes in each subtype. The findings might help predict the rate of nonparoxysmal AF progression and improve the efficacy of anti-inflammatory treatments in AF.

## Conclusions

This study revealed two major nonparoxysmal AF subtypes and eleven hub genes, which provide potential therapeutic targets for anti-inflammatory treatments of nonparoxysmal AF.

## Supplementary Information

Supplementary Information.

## Data Availability

The raw array data are available in the Gene Expression Omnibus database (GEO submission: GSE41177, GSE79768, GSE115574, and GSE31821).
