# Supplementary material for: Characteristics of immune clusters and cell abundance in patients with different subtypes of nonparoxysmal atrial fibrillation
Source: Sci Rep. 2023 Jan 18;13:968. doi: 10.1038/s41598-022-26749-z (PMC9849221; doi:10.1038/s41598-022-26749-z)

**Supplementary file**

Figure 1 Box plots for the expression data in all included samples. (A) Box plot of the raw expression data. (B) Box plot of the expression data after RMA normalization. (C) Box plot of the expression data after batch normalization.


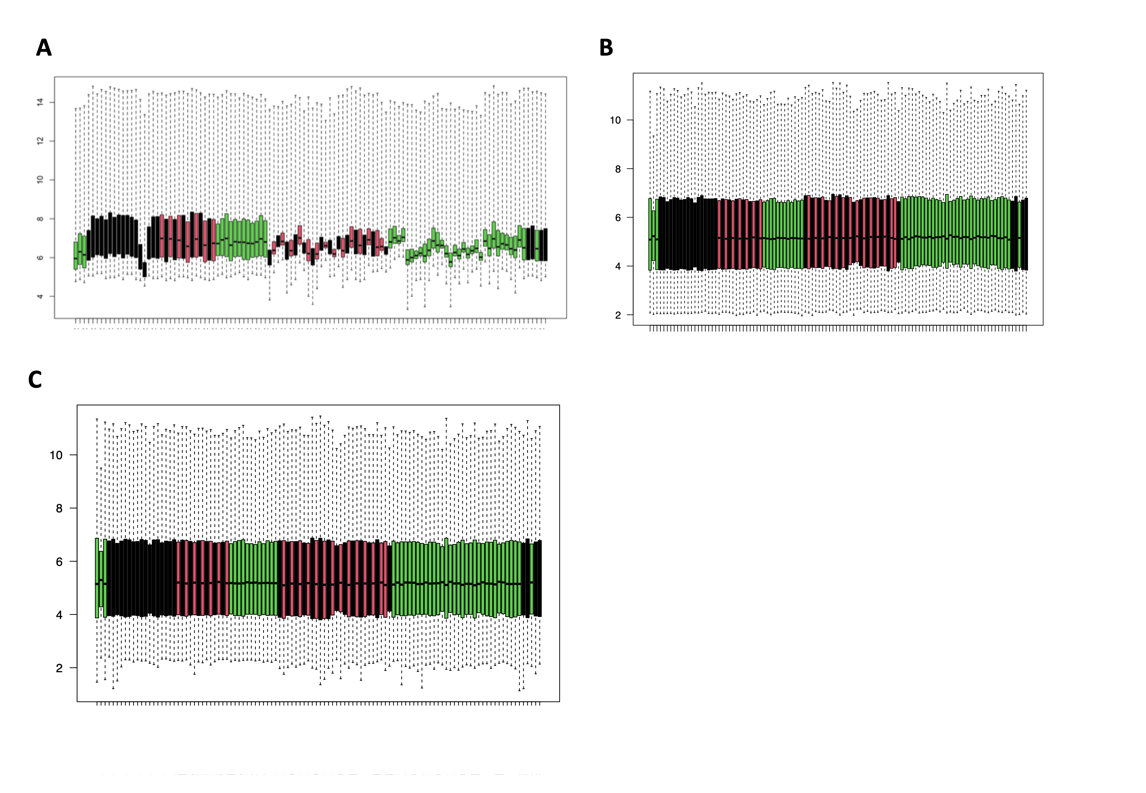


Figure 2 Immune cluster characterization and correlation analysis. (A) Immune cluster characterization histogram of all included samples estimated using ssGSEA. (B) Immune cluster characterization histogram of different groups estimated using ssGSEA. (C) ﻿Correlation matrix of the CIBERSORT results. ﻿Red dots represent positive correlations, and blue dots represent ﻿negative correlations. ﻿The size of the dot is positively associated with the correlation coefficient.
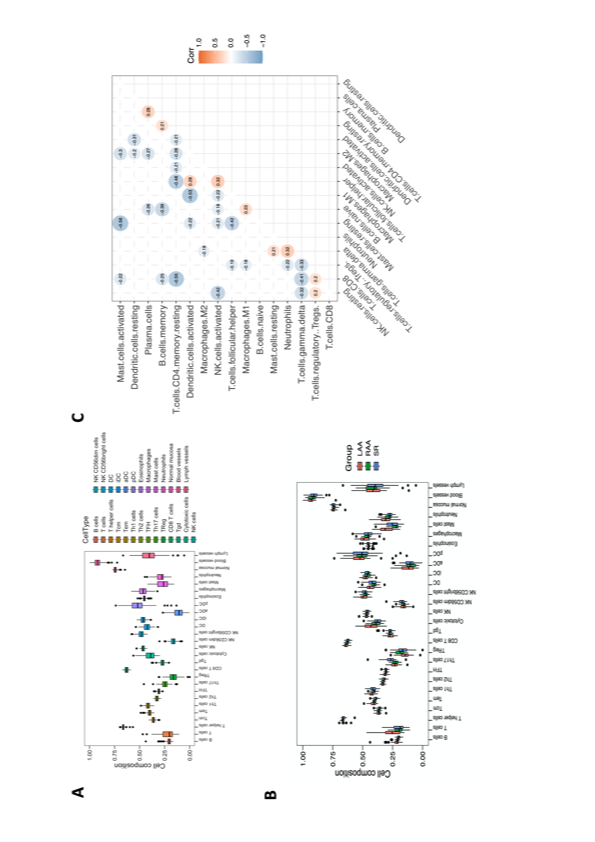


Figure 3 The volcano diagrams of differentially expressed genes. (A) LAA vs. SR volcano diagram. (B) RAA vs. SR volcano diagram. (C) LAA vs. RAA volcano diagram.


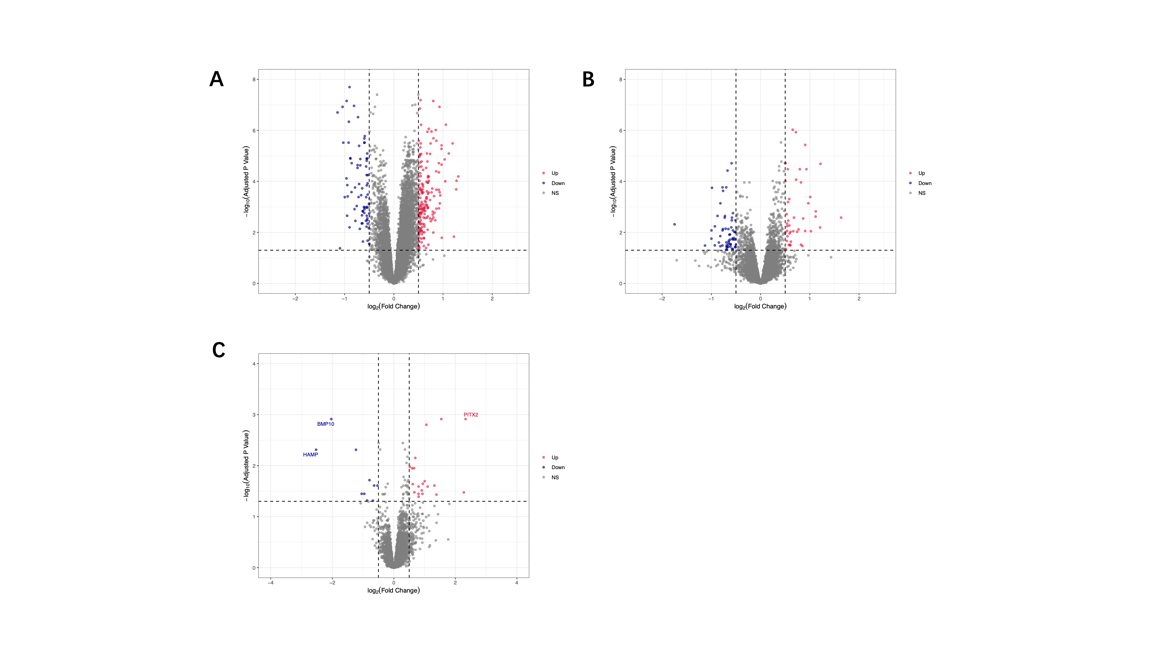


Figure 4 ﻿Enrichment analyses of the brown module in the WGCNA (‘clusterProfiler’ package). (A) The GO terms ﻿analysis for biological processes. (B) The GO terms ﻿analysis for molecular function. (C) The GO terms ﻿analysis for cellular components. (D) ﻿KEGG pathway enrichment analysis.


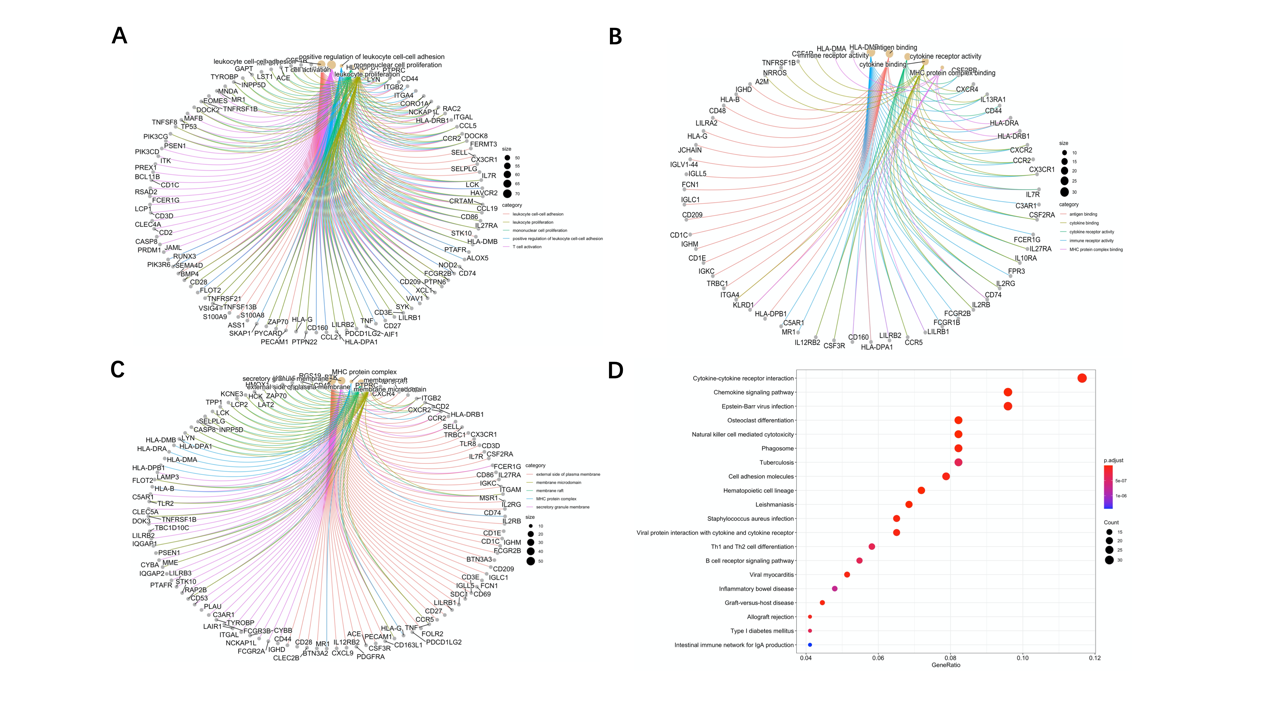

Supplement: Supplementary file 1 — Supplementary Information. [file 41598_2022_26749_MOESM1_ESM.docx]
